# Effectiveness of Montelukast on asthma control in infants: methodology of a French claims data study

**DOI:** 10.1186/s12890-015-0047-6

**Published:** 2015-05-02

**Authors:** Manon Belhassen, Gérard de Pouvourville, Laurent Laforest, Jacques Brouard, Jacques de Blic, Brigitte Fauroux, Valérie Laigle, Céline Chanut-Vogel, Liliane Lamezec, Eric Van Ganse

**Affiliations:** Merck Sharp & Dohme, Paris, France; Claude Bernard University, UMR CNRS 5558, Lyon, France; ESSEC, Paris, France; Pediatric Medicine University Hospital, Caen, France; Pediatric Medicine Necker University Hospital, Paris, France; Pediatric Noninvasive Ventilation and Sleep Unit, Necker University Hospital, Paris, France; Respiratory Medicine, Croix Rousse University Hospital, Lyon, France; RIPPS Network, Paris, France

**Keywords:** Asthma, Therapy, Control, Exacerbation, Infants, Effectiveness, Claims data, Cohort study, Pilot study

## Abstract

**Background:**

This pilot study, conducted on a 1/97th representative sample of French claims data, prepared a project to assess the effectiveness of Montelukast (MTL-4) as add-on therapy for asthma in infants (6–24 months) compared to inhaled corticosteroids (ICS), based on real-world data. Due to the very recent opening of French claims data for effectiveness research, and the complex structure of this data source, we first tested the feasibility of identifying infants with asthma and outcome criteria, and the ability to perform relevant comparisons.

**Methods:**

We identified a cohort of infants with uncontrolled asthma and receiving ≥2 consecutive dispensations of any respiratory drug (R03 ATC classification) during a 6-month period. Uncontrolled asthma was identified either from exacerbations or from markers of acute loss of asthma control; date of occurrence of an event (exacerbation and/or acute loss of asthma control) was defined as index date. The study groups comprised infants receiving MTL-4 +/− ICS (MTL-4 group) or ICS without MTL-4 (ICS group) at index date. These two groups were matched on gender, age, quarter of index date, therapy before index date, past asthma-related hospitalization (ever), and were followed for 6 months. The outcome was the rate of infants with uncontrolled asthma, defined as above.

**Results:**

This pilot cohort study included 1,149 infants with asthma (mean age 14.1 months, 64% boys). Of these, 51 and 768 were assigned to the MTL-4 and ICS groups, respectively. Uncontrolled asthma occurred in 78.8% and 78.4% of infants in these groups, respectively (oral corticosteroids were dispensed to 49% and 61%, respectively). Assessment of uncontrolled asthma, exposure to MTL-4 and ICS, and occurrence of outcomes were achieved. For the development of matching criteria, we defined a new marker of severity (therapeutic typologies).

**Conclusion:**

These data support the feasibility of the final project, to be conducted on claims data from the whole French population. We also showed that, with appropriate methodology and by using valid criteria, French claims data are an adequate resource for conducting comparative effectiveness studies in pediatric asthma. Finally, the algorithm used to identify infants with asthma could be applied to other studies using claims data.

## Background

Asthma is a common disease in childhood. In the European Union, it was estimated that 7% of children and adolescents have self-reported asthma and 12% wheezing [1]. Montelukast 4 mg (MTL-4) is an add-on therapy for young asthmatic children. MTL-4 was approved in March 2010 for use in children aged 6 months to 5 years with mild to moderate persistent asthma, insufficiently controlled with inhaled corticosteroids potentially associated with short acting beta agonists [2].

Regular therapy with inhaled corticosteroids (ICS) or leukotriene-receptor antagonists (LTRA) is effective in reducing asthma exacerbations in adults and children [3-6]. International guidelines recommend ICS as preferred controller medication in children with asthma, with LTRA as an alternative option [7-10]. This recommendation was based on a 2004 Cochrane Review of 27 trials including adults or children that reported a 65% higher risk of exacerbations requiring systemic steroids in patients treated with LTRA compared to those treated with ICS [11]. These trials entailed rigorous drug prescribing and closely monitored subjects, resulting in more regular drug use than is observed in clinical practice. In contrast, drug claims studies have revealed that similar or better health outcomes were obtained with LTRA compared to ICS, raising uncertainty as to the relative effectiveness of ICS versus LTRA in real-life practice [12,13].

Pharmacoepidemiological studies using administrative healthcare databases are a powerful means of assessing drug use and effects in clinical practice. National French claims data (Système National d’Information Inter-Régimes de l’Assurance Maladie, SNIIR-AM) record exhaustive reimbursed medical resource utilization, including related costs, for the overwhelming majority of the French population, i.e. 86% of 65.4 million people. However, due to the recent opening of SNIIR-AM data for effectiveness research, and the complex structure of this resource, the feasibility of our final project (which is to compare the effectiveness of MTL-4 vs. ICS on health outcomes of infants with mild to moderate uncontrolled asthma) was first tested here using a 1/97th sample of the full data set (Echantillon Généraliste de Bénéficiaires, EGB) [14].

The questions addressed by the EGB feasibility study included the identification of infants with asthma and the extent of MTL-4 and ICS use (to assess the statistical power for group comparison). We also verified the occurrence of outcomes (acute loss of asthma control and exacerbations) and the feasibility of matching according to predefined criteria.

## Methods

### Data source

SNIIR-AM records exhaustive medical resource utilization of the population covered by the national health insurance scheme, including hospital activity and expenditure data (Programme Médicalisé des Systèmes d’Information, PMSI). The EGB is a 1/97th representative random sample of the SNIIR-AM. It includes information on long-term disease status (LTD status) that allows severe patients to receive therapy without advancing payment. The EGB also contains information on free-access-to-care status (CMU-C) that enables patients of low socioeconomic status to receive free medical care. In the absence of diagnoses (except for hospitalizations or LTD status), French claims data usually require the use of proxies to identify asthma and outcomes criteria.

This observational study was conducted on anonymized claims data, and the National Informatics and Liberty Committee has delivered an overall authorization to use EGB data for research purposes. This study has been performed with the approval of the French institute of health data (Institut des Données de Santé, approbation n°37, February 1, 2012).

### Study design

**Figure 1:**
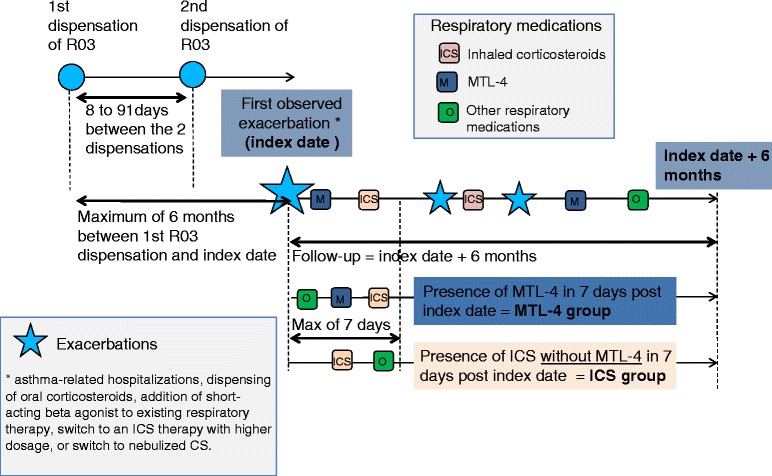
General study design.

Using EGB data, we constructed a prospective cohort of infants with asthma; two groups were identified, one treated with ICS (beclomethasone, fluticasone, or budesonide) without MTL-4, and the other with MTL-4 with or without ICS (Figure 1). In the French Guidelines [2], MTL-4 is indicated as add-on treatment for asthma in patients with mild to moderate persistent asthma, when they remain inadequately controlled by inhaled corticosteroids associated or not to short-acting beta-2 adrenergic agonists. To date, there is no indication in this age group to prescribed MTL-4 as controller in monotherapy.

### Study population

We selected infants:

- Considered to be asthmatic, i.e. receiving ≥ 2 consecutive dispensations of any respiratory drug (R03 ATC classification), from March 8, 2010 (launch of MTL-4 in France). The second R03 medication had to be dispensed between 8 and 91 days after the first. Infants had to be aged 6 to 24 months at dispensation of the first R03 drug.
- Presenting uncontrolled asthma, identified from exacerbation (i.e. asthma-related hospitalizations ICD-10 codes J45 or J46, or dispensation of oral corticosteroids), or acute loss of asthma control (i.e. addition of short-acting beta agonists to existing respiratory therapy, switch to higher dose ICS therapy, or switch to nebulized CS), within 6 months following the first R03 dispensation. For all patients, the date of exacerbation or marker of acute loss of control was defined as the index date.
- With ≥ 6 months of follow-up in the EGB after index date.

### Compared therapeutic strategies

Infants initiating a treatment with ICS monotherapy were compared to those having MTL-4 (with or without concomitant ICS therapy).

MTL-4 and ICS groups were defined at index date:

- MTL-4 group: infants receiving a dispensation of MTL-4 within 7 days after the index date with or without concomitant ICS therapy,
- ICS group: infants not receiving MTL-4 within 7 days after the index date, nor in the 3 months before the index date. ICS was investigated only when dispensed in monotherapy (i.e. no fixed dose combinations).

### Study outcomes

The outcome was the rate of infants with uncontrolled asthma, defined as above.

### Statistical analyses

Included infants were described, and profiles were identified according to dispensed therapy (type of R03, and number of dispensations).

To ensure comparability of both groups, each infant from the MTL-4 group was matched with one or more infants from the ICS group, using the following criteria:

- Age at index date (+/− 3 months)
- Gender
- Number of R03 drugs before index date
- Quarter of index date

We performed two computations of the number of subjects needed to show specific decreases in uncontrolled asthma rates under MTL-4 compared to ICS (Table 1). For instance, to show a 12% relative decrease of uncontrolled asthma under MTL-4 [15], with a power of 90%, we needed at least 4,210 infants in each group, i.e. at least 42 infants in the MTL-4 group of this pilot study.

**Table 1:** **Characteristics of infants (N = 1,149)**

	**Number**	**Percentage**
Males	733	63.8%
Age (mean, months)	14.1 (SD = 5.6)	
Any Long-Term Disease status*	47	4.1%
Long-Term Disease status n°14**	9	0.8%
Free-access-to-care status***	250	21.8%

*Long-term disease status enables patients suffering from a severe chronic disease, in need of expensive chronic therapy, not to pay in advance for treatments dispensed in pharmacies and to be fully reimbursed.

**Severe chronic respiratory failure.

***Free-access-to-care status enables patients with socioeconomic difficulties to receive free medical care.

All analyses were performed using SAS software, version 9.3 (SAS Institute Inc., Cary, NC, USA).

## Results

### Identification of infants with asthma

**Figure 2:**
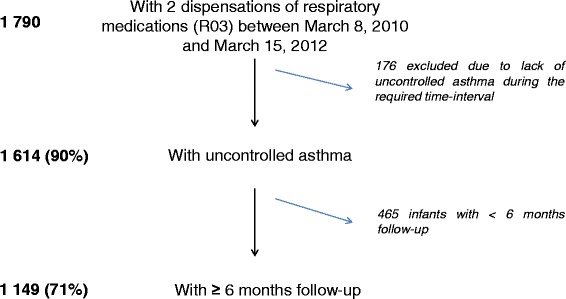
Patients’ selection.

In the EGB, we identified 1,790 infants meeting our definition of asthma (Figure 2).

Of these, 1,614 (90%) had uncontrolled asthma, according to our definition; 465 (26%) were not included due to limited duration of follow-up (less than 6 months after index date). Thus, our final sample included 1,149 infants with uncontrolled asthma.

The sample included a majority of boys (63.8%) (Table 1). Mean age was 14.1 months (SD = 5.6); 4.1% of infants benefited from LTD status, while 21.8% had free-access-to-care status.

### Frequency of exposure to MTL-4 and ICS

Of all infants meeting inclusion criteria, 51 (4.4%) and 768 (66.8%) were assigned to MTL-4 and ICS groups, respectively.

### Identification of outcomes (exacerbations and/or acute loss of asthma control)

After index date, uncontrolled asthma occurred in 78.8% of infants in the MTL-4 group and 78.4% of the ICS group. Oral corticosteroids were dispensed to 49% of the MTL-4 group and 61% of the ICS group, hospitalizations for asthma were identified in 3.9% and 4.8%, respectively, and acute loss of control occurred in 60.8% and 57.9%, respectively (Table 2).

**Table 2:** **Markers of uncontrolled asthma during 6-month follow-up (MTL-4 group, n = 51 and ICS group, n = 768)**

Exacerbation and/or acute loss of asthma control* (≥1)	**MTL-4 group n (%)**	**ICS group n (%)**
Markers of exacerbation:		
Dispensation of oral corticosteroids	25 (49.0%)	470 (61.2%)
Asthma-related hospitalizations	2 (3.9%)	37 (4.8%)
Markers of acute loss of asthma control**	31 (60.8%)	445 (57.9%)
Any of the above	40 (78.4%)	605 (78.8%)

*Not mutually exclusive; an infant could have one or more markers of exacerbation during the period.

**Addition of short-acting beta agonists to existing respiratory therapy, switch to higher dose ICS therapy, or switch to nebulized CS.

### Feasibility of matching, according to predefined criteria

For age, gender and quarter of index date, the distribution of the matching criteria in the two groups are described in Table 3, supporting the feasibility of matching according to these criteria.

**Table 3:** **Frequency of matching criteria**

**Matching criteria**	**MTL-4 group (n = 51)**	**ICS only group (n = 768)**	**MTL-4 + ICS groups (n = 819)**
Age (mean, months)	15.7	13.8	13.9
Female: n (%)	14 (27.5%)	286 (37.2%)	300 (36.6%)
Quarter of index date:			
Q1 2010-2011	9 (17.6%)	245 (31.9%)	254 (31.0%)
Q2 2010-2011	20 (39.2%)	221 (28.8%)	241 (29.4%)
Q3 2010-2011	10 (19.7%)	106 (13.8%)	116 (14.2%)
Q4 2010-2011	12 (23.5%)	196 (25.5%)	208 (25.4%)

Due to the limited number of R03 dispensations before index date (data not shown), and in agreement with the study Scientific Committee, the criterion “number of R03 before index date” was replaced with “pre-inclusion therapeutic typologies”, that was defined as follows:

- No specific treatment for asthma, or oral corticosteroids
- Reliever therapy only
- Controller therapy

## Discussion

This pilot study was performed using the EGB database, a 1/97 sample of the whole French population, to prepare a project aimed at assessing the effectiveness of MTL-4 in infants suffering from asthma. The first three questions addressed by this pilot study were answered satisfactorily, i.e. identification of infants with uncontrolled asthma (N = 1,149), frequency of exposure to MTL-4 and ICS (n = 51 and 768, respectively), and occurrence of outcomes (78% and 79% of MTL-4 and ICS infants, respectively). For the last question, i.e. development of matching criteria, we defined a new marker of severity (therapeutic typologies).

Asthma in infants under 36 months is usually defined as an episode of wheezing dyspnea that has occurred at least 3 times since birth [2]. In our study, as diagnoses are usually missing in French claims data, the identification of asthmatic infants relied on a proxy, i.e. the use of specific therapy (2 consecutive dispensations of respiratory drugs ATC R03, separated by an interval of 8 to 91 days). Based on this approach, 1,790 asthmatic infants were identified between March 2010 and March 2012. Combined with the mean annual number of births in France – around 790,000 –, the prevalence of asthma may be estimated to be around 11% in infants. Epidemiological data on the prevalence of asthma in infants are rare. One report suggests that the prevalence of these symptoms at the age of two lies between 11.9% and 26.6% [16], which is compatible with our results. The sex ratio observed in our study is consistent with data available for young children under 5 years, where a 2/1 ratio is traditionally observed in favor of boys [17]. In summary, prevalence and sex-ratio support the validity of the algorithm used for the identification of infants with asthma in our study.

Using our selection criteria, 51 users of MTL-4 were identified in the EGB, together with 768 users of ICS. These represent adequate counts to meet the objectives of the final study, in particular for group comparison. Sales data suggest that a maximum of 78,000 infants aged 6 to 24 months were exposed to MTL-4 over a 3-year period (2010 to 2012), which corresponds to around 250 infants/year in the EGB, in line with our figures stemming from strict inclusion criteria [18]. Similarly, the identification of outcomes in the EGB and their frequency also confirmed that the study was feasible using claims data (≥49% of infants in the MTL-4 and ≥57.9% of the ICS groups were identified with uncontrolled asthma, or dispensing of oral corticosteroids). According to the IRDES (Institut de Recherche et Documentation en Economie de la Santé, the national health economics research institute) survey [17], in 60% of patients, asthma is inadequately controlled (based on GINA criteria). According to stricter criteria from the French health authority (Haute Autorité de Santé, HAS) guidelines [2], asthma is inadequately controlled in 83% of patients. In the Er’Asthme study [19,20] that investigated children aged 6 to 14 years, considering the Canadian criteria which were similar to HAS criteria, asthma was inadequately controlled in 73% of children. The results obtained in our explanatory study are not incompatible with these references, again supporting their validity.

This pilot study was conducted using the EGB database [14], which is a sample of French national claims data. In several countries, comparative effectiveness research studies have been conducted in asthma using claims data [21,22]. In France, our study was among the firsts with this objective, justifying a feasibility study. A major advantage of EGB is to record exhaustive medical resource utilization in a 1/97th sample of the 86% of French population covered by the national health insurance system. In addition to detailed data on drug utilization, the EGB also contains data on all medical procedures, and all medical and paramedical visits. Information on prescribers is also available. The linkage of primary care with hospital data (PMSI) provides access to data on hospitalizations (private and public hospitals), i.e. exhaustive individual medical resource utilization. As the SNIIR-AM records health care provided to infants following exposure to MTL-4, it was *a priori* considered to be a useful source of data to test the effectiveness of this drug. Another approach would have been a field study in a population of asthmatic infants. Besides practical pitfalls, such as complex organization and long timelines, a field study would have been exposed to recall bias, particularly on past medical resource utilization.

Nonetheless, the EGB database has certain intrinsic limitations. Few variables were available to describe patients: age, gender, free-access-to-care status, and LTD status in case of more severe disease. EGB does not include clinical data (e.g. lab test results), or information on family history. Prescriptions without dispensations or dispensations without drug utilization by the patient are not recorded, neither are prescribed doses or durations of prescriptions [23].

This study had other limitations. Uncontrolled asthmatic infants were identified using ≥2 distinct R03 drug dispensations followed by exacerbation and/or acute loss of control. These criteria excluded a significant proportion of MTL-4 -exposed infants. This also limited the generalizability of our findings. For example, the rate of outcomes during the 6 months of follow-up could have been overestimated by the choice of the selection criteria, as asthma had to be uncontrolled for infants to be included. Also, defining exacerbations from dispensations of oral corticosteroids could be criticized as such use could be due to conditions other than asthma. However, it is likely that requiring the initial dispensation of oral corticosteroids to be followed within seven days by a dispensation of MTL-4 or ICS ensured adequate specificity of this marker to identify asthma exacerbations.

Finally, the matching for asthma severity was based on proxies (therapeutic typologies and past asthma-related hospitalization) and probably did not fully account for potential differences in severity between the two groups. Therapeutic typologies allowed us to classify infants into three groups of severity based on treatments before index date: infants with no asthma treatment, infants with only reliever therapy, and infants already receiving controller therapy. We believe that this classification optimized the use of information available in French claims data.

Importantly, the study design was based on the assumption that uncontrolled asthma leads to increased medical resource utilization, and this concept is now generally accepted, as exemplified by recent definitions [2,24]. Other methodological biases are, however, possible. As in any observational study comparing effectiveness between two therapeutic strategies, indication bias can never be excluded [25]; nonetheless, as already stressed, matching of infants on the typology of initial therapy limits this bias.

## Conclusions

Our findings support the feasibility of a final SNIIR-AM project for the identification of asthmatic infants, their outcomes, and matching criteria; this allowed us to finalize the SNIIR-AM protocol. In addition, this study confirmed that, with appropriate methodology and by using valid criteria, French claims data are an adequate resource to conduct comparative effectiveness studies in asthma and could be a practical alternative to field studies. From national claims data, the prevalence of asthma in France was estimated to be around 11% in infants. Finally, the algorithm used to identify infants with asthma could be applied to other studies using claims data.
